# Connectivity Mapping for Candidate Therapeutics Identification Using Next Generation Sequencing RNA-Seq Data

**DOI:** 10.1371/journal.pone.0066902

**Published:** 2013-06-26

**Authors:** Darragh G. McArt, Philip D. Dunne, Jaine K. Blayney, Manuel Salto-Tellez, Sandra Van Schaeybroeck, Peter W. Hamilton, Shu-Dong Zhang

**Affiliations:** Centre for Cancer Research and Cell Biology(CCRCB), Queen's University Belfast, Belfast, United Kingdom; Justus-Liebig-University Giessen, Germany

## Abstract

The advent of next generation sequencing technologies (NGS) has expanded the area of genomic research, offering high coverage and increased sensitivity over older microarray platforms. Although the current cost of next generation sequencing is still exceeding that of microarray approaches, the rapid advances in NGS will likely make it the platform of choice for future research in differential gene expression. Connectivity mapping is a procedure for examining the connections among diseases, genes and drugs by differential gene expression initially based on microarray technology, with which a large collection of compound-induced reference gene expression profiles have been accumulated. In this work, we aim to test the feasibility of incorporating NGS RNA-Seq data into the current connectivity mapping framework by utilizing the microarray based reference profiles and the construction of a differentially expressed gene signature from a NGS dataset. This would allow for the establishment of connections between the NGS gene signature and those microarray reference profiles, alleviating the associated incurring cost of re-creating drug profiles with NGS technology. We examined the connectivity mapping approach on a publicly available NGS dataset with androgen stimulation of LNCaP cells in order to extract candidate compounds that could inhibit the proliferative phenotype of LNCaP cells and to elucidate their potential in a laboratory setting. In addition, we also analyzed an independent microarray dataset of similar experimental settings. We found a high level of concordance between the top compounds identified using the gene signatures from the two datasets. The nicotine derivative cotinine was returned as the top candidate among the overlapping compounds with potential to suppress this proliferative phenotype. Subsequent lab experiments validated this connectivity mapping hit, showing that cotinine inhibits cell proliferation in an androgen dependent manner. Thus the results in this study suggest a promising prospect of integrating NGS data with connectivity mapping.

## Introduction

The next generation of sequencing technologies are expanding our capabilities in modern cancer research. NGS offers such advantages over the older constrained microarray approach in increased sensitivity, not suffering from cross hybridisation and the fact that no dependence on any prior knowledge is necessary, as have been discussed in other articles [1]–[7]. The measurement of the transcripts by this technique, RNA-Seq, has been steadily growing as a method in recent years. The technique provides a wealth of information on a cellular state and biological insight can be obtained using appropriate pipelines for analysis [2], [6], [8]. The millions of short reads from reverse transcribed RNA generated in this process are sheared, and perhaps size selected, into measurable strands of cDNA where ligated adapters are attached for sequencing in single or paired-ends depending on the experimental question [2].

The current sequencing platforms utilize different technologies to try and achieve the same end goal with machines from Roche, Illumina and Life technologies (plus arriving soon will be Ion Torrents Proton) allowing for RNA-Seq analysis with sufficient coverage [9]. The resulting output should be millions of reads of data from 25 to 300 base pairs [2]. The typical process for NGS is to align the millions of reads to a reference genome/transcriptome, this reference can be supplemented with particular filtered libraries. The aligners tend to fall within two categories, those of Burrows Wheeler transform (such as BWA or BOWTIE) based approach or those of a hash table (such as SHRIMP or SOAP) based approach [10]–[13]. The choice of aligner is largely down to performance versus complexity issues and have been addressed before [1], [14]. Once aligned or 'mapped' to a genome the reads are normally summarised and sorted or indexed to speed up performance followed by a normalization step to allow sample expression comparisons utilising differential expression where new methods are arising all the time [15].

Marioni et al. performed tests to compare the ability of microarray technologies with that of the NGS method for measuring steady state RNA to identify differentially expressed genes [16]. They performed their analyses on liver and kidney RNA samples and noted the RNA-Seq's ability to perform with little technical variation. They suggested at least an 81 percent overlap between differentially expressed genes between platforms. The RNA-Seq method, they furthered, offered more often than not the true positives when the platforms differed, validated in a laboratory setting by qPCR for a selection of genes declared differentially expressed on one platform. They also found that the RNA-Seq approach was highly reproducible and required fewer technical replicates. Recently, Su et al. furthered this by examining the comparison of a microarray platform with an NGS platform for the same set of toxicological samples. They found similar gene expression profiles for both platforms with RNA-Seq more sensitive at detecting low expressing genes. They found the overlap of differential expression at upwards of 50 percent between the two platforms but that RNA-Seq and microarray maintained a consistent biological interpretation [17], with some of the non comparable differential gene expression findings attributed to RNA-Seq's higher dynamic range in detection. RNA-Seq analysis pipelines are being continually updated and evolving to the state where EBI have supplied an extremely useful resource in an Rcloud, including a tool ArrayExpressHTS [18], which is very flexible in user choices.

Connectivity mapping is a bioinformatic technique to make connections between disease, genes, and drugs and has been implemented since 2006 by Lamb et al. [19] providing a valuable resource which has also been successfully exploited with different approaches and for different purposes, eg, for the construction of a drug similarity network [20], or for assessing the regulatory activity of a drug on its target genes [21]. The fundamental basis of a gene expression connectivity map is the building of large scale reference profiles that can be utilised against a signature gene set that would best characterise the difference between two cellular states. The reference profiles themselves are curated data based on a particular drug - dose - cell line microarray analysis. The volume of these reference profiles have increased vastly to over 6000, providing an attractive database of compound-induced gene expression profiles against which query gene signatures can be compared. The hit compounds from connectivity mapping can be ranked by an appropriate scoring metric to suggest candidate therapeutics for the particular disease state from which the signature was derived. sscMap was developed in 2008 by Zhang and Gant [22]–[24] and represents an attractive model of the connectivity mapping process. The technique has the added statistical stringency to guard the results against false positives in the analysis. A recent review on drug repositioning by Iorio et al. describes the development in matching gene expression signatures in order to connect phenotypes and the role of connectivity mapping in reverting undesirable phenotypes. They suggested that RNA-Seq may be an attractive approach to overcome the limitations of microarray technology by having a large dynamic range, as microarrays do not measure gene expression in absolute units [25].

**Figure 1:**
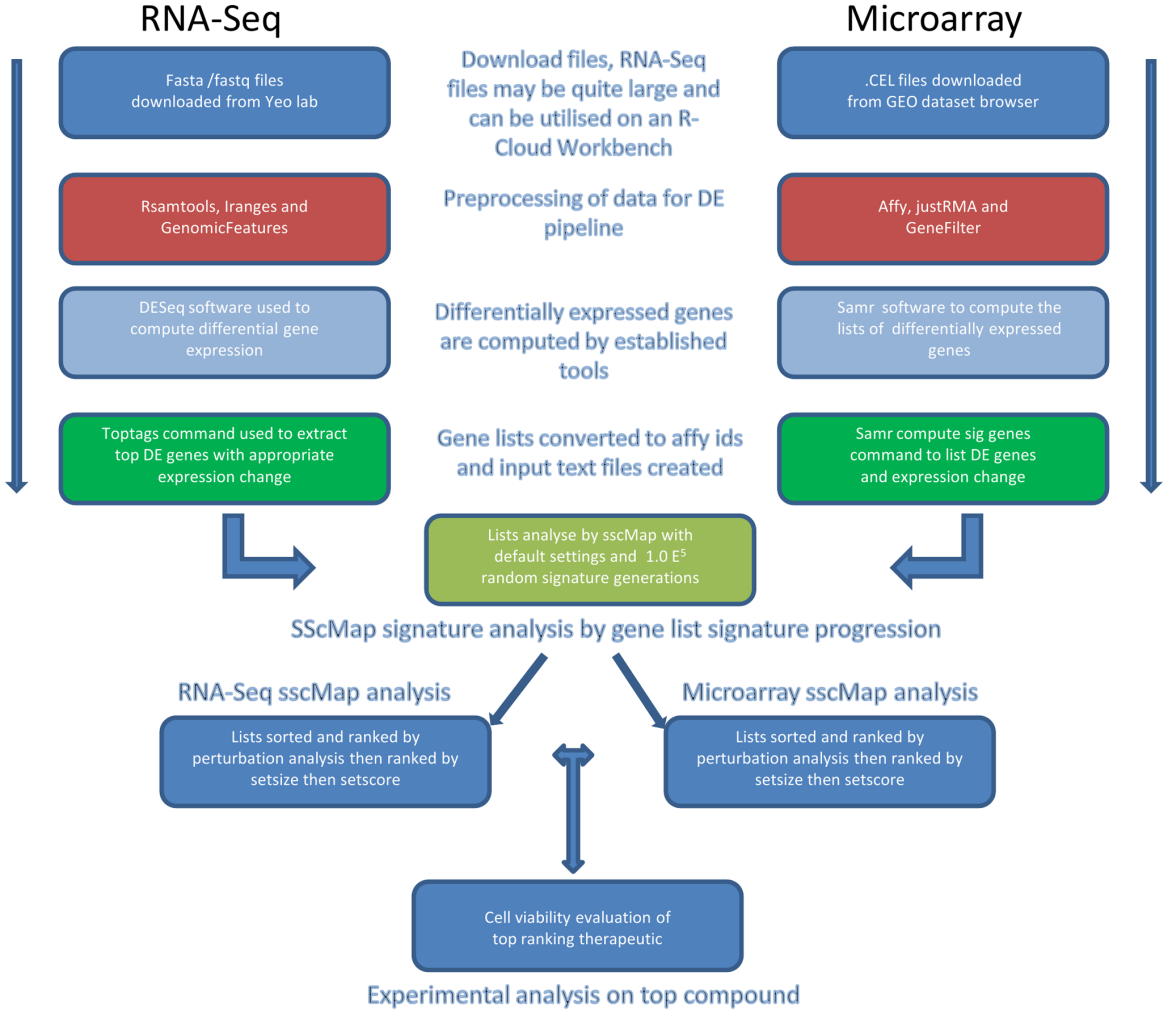
Flow chart of processing stages involved in establishing signatures from RNA-Seq and Microarray analysis for connectivity mapping.

With the ability to obtain a list of differentially expressed genes from both platforms for analysis, connectivity mapping may be able to continue in the immediate future unabated until such a time as when it becomes economically and scientifically viable to create NGS reference profiles. Since essentially all that is required for connectivity mapping is a list of the top ranked differentially expressed genes, this can be obtained from an approach such as in RNA-Seq analysis. Utilising an established analytic method [2] we examined the possibility of analysing an established experiment obtained from published fasta files. The data were in the format of single end reads from an Illumina platform. The dataset pertained to an androgen sensitive prostate cancer model by Li et al. [26]. In order to make sure the therapeutic candidates were robust against minor signature agitations, we applied the gene signature perturbation method described previously [24]. This allows the ranking of candidate compounds according to their ability to withstand subtle changes and make them more reproducible between researchers. We compared the results from RNA-Seq gene signature against that from an experimentally similar microarray dataset [27], and tested the top hit in a laboratory setting. Figure 1 summarizes the key processing and integration steps we followed in this study. This novel approach to analysing RNA-Seq data will in no doubt be a highly desired approach in cancer research where potential therapeutics are sought for cancers with poor prognosis.

## Materials and Methods

### RNA-seq Dataset

The Li et al. prostate cancer dataset was directly obtained from the Yeo laboratory website ( [26], http://yeolab.ucsd.edu/yeolab/Papers.html). It contained seven Illumina samples S1–S7 which represented four untreated and three treated samples of androgen-sensitive human prostate adenocarcinoma LNCaP (Lymph Node Carcinoma of the Prostate) cells which were androgen stimulated. These sequences did not contain any quality scores with the underlying sequence. The data contained within were 35 nucleotides in length and single-ended reads. RNA-Seq analysis was performed as described by Oschlack et al. [2]. Briefly, the tools are established for extraction of genes based on a Negative Binomial model for differential expression. The aligner used was BOWTIE (version 0.12.7) [10], with the reference genome ‘hg19’ downloaded from University of California, Santa Cruz(UCSC) database [28]. This was extracted and stored in an folder located by BOWTIE_INDEXES on a Linux machine. The standard reference was used for analyses with no filtered databases applied as this would be surplus to the connectivity mapping procedure. BOWTIE alignments were run with the -best tags for the single-end reads and -v3 command as it did not have quality scores with the reads. The -f tag was also used as the files were in fasta format. The dataset was run with -p4 and -sam command to allocate four threads in the analysis stage and to obtain SAM outputs, all other commands were left as standard.

Output SAM files containing the aligned reads are then converted into BAM files using the samtools (version 0.1.8) software with commands used to import, sort and index the files in BAM format [29]. This lessens the memory footprint required when using downstream analysis methods such as the R statistical package for discovering differential expression. R is freely downloadable software (http://www.r-project.org/) containing many peer reviewed packages that can be used in different biological statistical analyses.

The computational requirements to analyse RNA-Seq data are intensive. The Li et al. prostate cancer study was performed on an R-Cloud. The R-Cloud Analysis is an attractive avenue for datasets that are particularly large and is offered by the European Bioinformatics Institute. The EBIs R-Cloud allows up to 64Gig computing servers for analysis of particularly large datasets under an R environment. The platform offered ease of use and strong technical support (http://www.ebi.ac.uk/Tools/rcloud/). The resulting BAM files were uploaded to the R-Cloud (version 1.1.1) and imported into a dedicated 32Gig server (BENCH
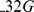
_19 Tau) and the commands could be performed as if on a local machine. Initially in R, we used the Bioconductor package Rsamtools to obtain an interface for the BAM files created. This is used with other R Bioconductor packages [30] (such as IRanges and GenomicFeatures) that can be used to manipulate the BAM files. GenomicFeatures has three classes (GRanges, GRangesList, and GappedAlignments) and is used to represent the genomic locations. In the datasets analysed the GRanges class was set as ambiguous for the strand designator. From here, the BAM files are analysed by GenomicFeatures. Briefly, this package retrieves and manages transcript-related features which utilises the RNA-Seq data with resources from UCSC Genome Bioinformatics and BioMart. It creates a 'TranscriptDb' object to store transcript metadata and in this study the 'makeTranscriptDbFromUCSC' command was used on the 'hg19' genome with the supported track of Ensembl genes. Further to this we use the 'transcriptsby' command to maintain the relationships of the transcripts to a biological context, here we use 'transciptsBy' with the type of feature grouping factor 'gene'. The locations and identifiers are now contained in a GRangesList. The 'countOverlaps' function contained in the GenomicRanges package can now be used to count the overlaps for each read in the query. With the data summarized into a table of counts we then used DESeq to create our list of differentially expressed genes as DESeq is good at small sample sizes by borrowing strengths from closely related genes statistically, first estimating sizefactors and then estimate dispersions followed by the negative binomial test and order the results [31]. As a comparison to the results of DESeq, EdgeR was also used to analyse the data to obtain lists of differentially expressed genes utilizing the TMM method to supply appropriate scaling factors and then this is incorporated into the DGEList with an 'estimateCommonDisp' method applied followed by exactTest [32], which is a generalization of the exact binomial test. The topTags command was used to extract the top differentially expressed genes with a chosen level of statistical significance.

### Microarray

Microarray data were downloaded from a study by Wang et al [27] (GEO Accession Number GDS3111), which looked into a hierarchical network of transcription factors that would govern androgen receptor-dependent prostate cancer growth. There were nine files in this study which were downloaded from GEO by the Accession GSE7868 in zipped format into a folder and extracted. This contained nine CEL files which encapsulated three replicates of increasing time exposure to androgen stimulation by DHT over 0, 4 and 16 hrs. Initial analysis was carried out using GEOs Dataset Browser which allowed graphical representation of the genes in the list [33], [34]. Analysis of the data was carried out using R packages SamR(v2.0) [35], affy(v1.34.0) [36], and genefilter(v1.38.0) [37] with their associated dependencies. The nine files were analysed by affy's justRMA() and their expression status extracted. We then used the package genefilter to remove the genes with small variance across all the files by filtering from the median. With the small size in samples we utilised the package SamR to extract differentially expressed genes. The differential groups were 0 hrs and 16 hrs, using a median FDR threshold of 0.05 obtained by two class unpaired test, random seed and 100 permutations. Genes from high and low tables were copied to Microsoft Excel for inspection and formulation of a gene signature for connectivity mapping.

### sscMap

Connectivity map analysis was performed using the sscMap software [22], [23], which is a stand alone Java application running across different Operating Systems. The connectivity mapping approach requires three key components: query gene signature, reference profile database, and pattern matching algorithm. At the heart of the original CMap and later sscMap, is a core database of reference gene expression profiles derived from large scale systematic microarray experiments by the Broad Institute. The current release of the Broad Institute Connectivity Map (Build 02) (http://www.broad.mit.edu/cmap/) contains over 6000 individual reference profiles, and so does the sscMap, whose core database of reference profiles was created using the same raw microarray datasets. Details on the procedures and guiding principles of constructing reference profiles can be found in papers that introduced the frameworks, for CMap [19] and for sscMap [22], [23], respectively. The sscMap application with gene signature perturbation capacity can be freely downloaded from ftp://ftp.qub.ac.uk/pub/users/sdzhang/perturbation. Bundled with the downloads are detailed description and guided tours on how to use the software and interpret the results. The query gene signatures used in this study are derived from the results of differential expression analysis on the RNA-seq data and microarray data. The differentially expressed list of genes returned by topTags from DESeq were mapped to Affymetrix HG-U133A Probeset IDs before feeding to sscMap. An assortment of signature sizes were run in sscMap with a large number (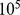) of randomization and permutations conducted to gauge statistical significance. These lists served as a bench mark to find an optimal size of the gene signature where a FDR threshold (0.01) is met with the minimum number of genes. The gene signature with optimal size, 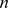, was then run again in sscMap with the gene signature perturbation procedure. This allows us to measure the stabilities of the discovered connections by their ability to withstand a series of single gene omission with replacement. The candidate compounds that withstood these perturbations received a score quantifying their perturbation stability [24]. We also carried out connectivity mapping analysis on the microarray data, taking as input the differentially expressed genes detected by SamR between the time points 0 hrs and 16 hrs. As this microarray dataset was based on Affymetrix HG-U133 Plus 2.0 microarray platform, we filtered out Probeset IDs that are not part of HG-U133A arrays before feeding the gene signature to sscMap. This list of DEGs were processed in the same fashion as described above.

### GeneCodis Analysis

The gene lists obtained from both the NGS gene signature and the microarray gene signature were combined and submitted to GeneCodis [38], [39], an on-line modular enrichment tool. GeneCodis assesses if an input list of genes results in combinations of annotations that are significantly enriched. For this analysis, the following annotations were selected: GO Biological Process, GO Molecular Function, GO Cellular Component, KEGG Pathways, InterPro Motifs, Panther Pathways and Transcription Factors. Of particular interest were processes and pathways in which genes identified from both the NGS and microarray analyses participated in.

### Laboratory Analysis

#### Materials

Cell lines were maintained in RPMI media containing 10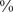 FBS and cultured at 37.0°C in a 5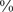 carbon dioxide incubator under aseptic conditions. Cotinine (Cat 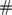 C5923) was purchased from Sigma (Dorset, UK), dissolved in ethanol, aliquoted and stored at −20°C.

#### Proliferation assay

Cells were seeded in triplicate at 50,000 cells per well in 6-well plates and allowed to attach overnight. Media containing the appropriate concentration of cotinine was added to each well. Fresh media with cotinine was replaced after 48 hours. Cell counts were carried out at 96 hours post treatment. Following treatment cells were trypsinised and resuspended in equal volumes of growth medium. 500
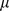
l of cell suspension was diluted with 100
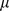
l of 0.1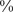 trypan blue staining solution (Sigma) and allowed to incubate at room temperature for 5 minutes. Viable cells were then counted using a hemocytometer. Viable cell counts for each treatment arm were plotted as viable cell counts relative to the control. Error bars represent standard deviation of counts in triplicate.

#### Cell doubling time assay

Cells were seeded in triplicate at 20,000 cells per well in 16 well xCELLigence E-plates from Roche and run on the xCELLigence system which provides real-time cell numbers across a give time frame. Specified drug treatments were carried out as for cell proliferation assay. The relative cell doubling time was calculated using the in-built software. Cell proliferation and cell doubling experiments were carried out 3 times independently using different cell stock batches.

#### Statistical analysis

The unpaired two-tailed t-test was used to determine statistically significant differences between treatment effects using Prism Graphpad software. 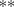 denotes p 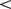 0.01.

## Results

### Differential Expression and sscMap Analysis

**Figure 2:**
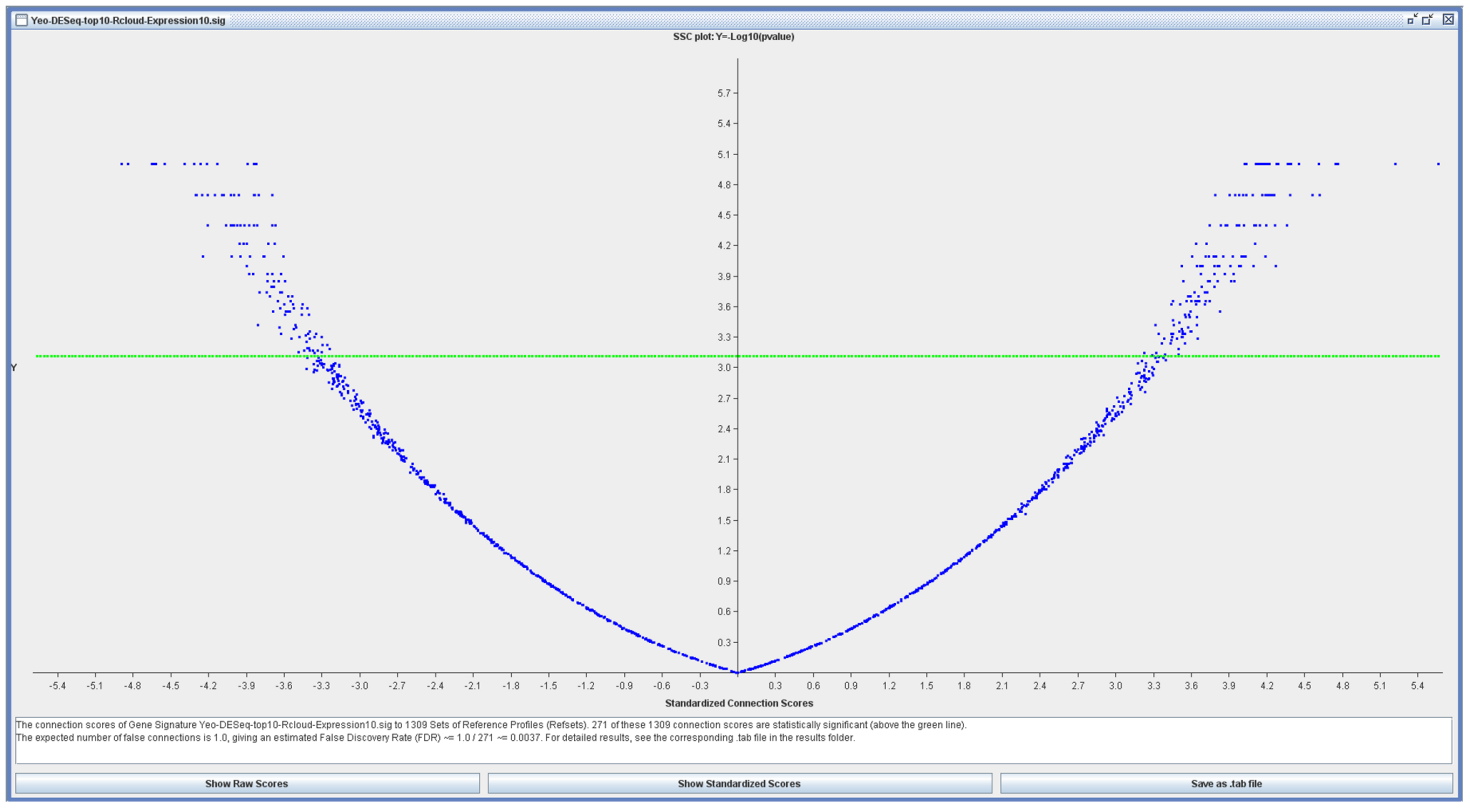
sscMap output for the signature from the RNA-Seq dataset. Figure demonstrates the volcano plot of the distribution of candidate compounds that may enhance (right side) or suppress (left side) the phenotype. Significant candidates are above the green line.

Table 1 and 2 list the top 10 differentially expressed genes returned from DESeq and EdgeR on the same RNA-seq dataset from the Yeo laboratory [12]. For the full list of differentially expressed genes returned by the DESeq and EdgeR analysis, please see Table S1 and Table S2, respectively. The overlap between the two top-10 lists is 9 out of l0. DESeq and EdgeR are both popular tools for differential expression analysis on RNA-seq data. Our results here suggest that their agreement is very high as far the top selected genes are concerned. Therefore, in subsequent sscMap analysis we chose the list of differentially expressed genes from DESeq to make query gene signatures, as DESeq was shown to make more balanced selection of differentially expressed genes throughout the dynamic range [31]. In connectivity mapping our focus is on the top selected genes as they will be used to form the query gene signature as input to sscMap. DESeqs top ranking genes were extracted and converted to Affymetrix Probeset IDs that would be usable in the sscMap software. The optimal signature size for sscMap is obtained by increasing the signature size until a set of statistically significant connections with an FDR 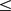
1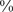 are first returned. In the current case of the RNA-seq data set, a gene signature with the top 10 mapped Affymetrix Probeset IDs (listed in Table 3) was determined to be an optimal length. Table 3 lists these 10 probeset IDs in the gene signature, and also shows the positions of these genes in the results of SamR analysis on the microarray dataset. Once acquired this gene signature was fed to sscMap with the gene signature perturbation procedure, which would check all the candidate compounds for their robustness against single gene omission. With this we can rank/prioritize the candidate compounds by their statistical significance, perturbation stability and their replicate number in the reference profile database. The criteria involved in order to fine filter the candidates was first by whether the p-value was significant with sscMaps Bonferonni correction, then if these candidates were significant by their perturbation stability which resided between zero to one. A perturbation stability score of one would indicate that the candidate compound remained significantly connected to the gene signature during all the perturbations. The list of mapped Affymetrix Probeset IDs in Table 3 represent seven different genes. This gene signature returned 271 compounds with statistically significant connections to it (Figure 2), of which 122 had a perturbation stability of 1 with which we would then sort by their setsize, which is the number of replicate reference profiles in the database for this compound. For equal setsizes, the compounds would then be sorted by their setscore to the gene signature. A negative setscore indicates that the compound is inversely connected to the gene signature, and may be useful to inhibit the phenotype represented by the signature. The top 2 ranking compounds in the list was haloperidol with a setsize of 32 and a setscore of 0.228 and genistein with a setsize of 17 and a setscore of 0.353. sscMap predicts that these compounds to have a high probability in enhancing the phenotype. The top candidate compounds that sscMap predicts could suppress the phenotype are nifedipine with a setsize of 7 and a setscore of −0.297 and cotinine with a setsize of 6 and a setscore of −0.598.

**Table 1:** DESeq top ranking differentially expressed genes.

EnsemblID	GeneSymbol	Mean-stimu	Mean-unstimu	ratio(stimu/unstimu)	log2Ratio	pvalue	adjustedPvalue	DSeqPosition	affy Mapped ID
ENSG00000151503	NCAPD3	3725.06	78.04	47.73	5.58	0.00E+00	0.00E+00	1	212789 at
ENSG00000096060	FKBP5	1605.85	47.32	33.94	5.08	0.00E+00	0.00E+00	2	204560 at
ENSG00000116133	DHCR24	1997.42	192.42	10.38	3.38	0.00E+00	0.00E+00	3	200862 at
ENSG00000156689	GLYATL2	1652.52	157.72	10.48	3.39	1.83E-317	1.47E-313	4	not Found In Annotation File
ENSG00000113594	LIFR	1030.64	54.44	18.93	4.24	7.33E-311	4.72E-307	5	205876 at
ENSG00000166451	CENPN	752.57	22.15	33.98	5.09	3.96E-299	2.12E-295	6	219555 s at, 222118 at
ENSG00000115648	MLPH	2733.96	422.61	6.47	2.69	2.81E-286	1.29E-282	7	218211 s at
ENSG00000244324	RP11-67L3.6	884.95	73.73	12.00	3.59	7.97E-235	3.21E-231	8	not Found In Annotation File
ENSG00000116285	ERRFI1	642.06	33.44	19.20	4.26	1.44E-226	5.14E-223	9	not Found In Annotation File
ENSG00000130066	SAT1	1049.43	138.12	7.60	2.93	3.58E-197	1.15E-193	10	213988 s at, 210592 s at, 203455 s at

The top 10 genes that were retrieved by DESeq using the R-Cloud on EBI for the LNCaP dataset. Expression ratio is (stimulated/un-stimulated). See Table S1 for the full list of differentially expressed genes returned by the DESeq analysis.

**Table 2:** EdgeR top ranking differentially expressed genes.

EnsemblID	GeneSymbol	log2Ratio	adjustedP	EdgeRposition	affy Mapped ID	DSeqPosition
ENSG00000151503	NCAPD3	5.58	0	1	212789 at	1
ENSG00000096060	FKBP5	5.10	0	2	204560 at	2
ENSG00000166451	CENPN	5.09	0	3	219555 s at, 222118 at	6
ENSG00000113594	LIFR	4.24	0	4	205876 at	5
ENSG00000244324	RP11-67L3.6	3.60	0	5	not Found InAnnotation File	8
ENSG00000156689	GLYATL2	3.40	0	6	not Found InAnnotation File	4
ENSG00000116133	DHCR24	3.38	0	7	200862 at	3
ENSG00000155368	DBI	3.01	0	8	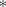 (202428 x at, 209389 x at, 211070 x at)	22
ENSG00000130066	SAT1	2.93	0	9	213988 s at, 210592 s at,203455 s at	10
ENSG00000115648	MLPH	2.72	0	10	218211 s at	7

The top 10 genes that were retrieved by EdgeR using the R-Cloud on EBI for the LNCaP dataset. Expression ratio is (stimulated/un-stimulated). Here we can see that the same set of identifiers used in the sscMap from the DESeq analysis would have been attained by EdgeR with the exception of ENSG00000155368 which was ranked 22nd in DESeq analysis. Table S2 contains the full list of differentially expressed genes returned by the EdgeR analysis.

**Table 3:** The gene signature from the NGS dataset using DESeq analysis and their positions in the microarray DEGs by SamR.

ProbeSetID	GeneSymbol	EnsemblID	log2Ratio	adjustedPvalue	DESeqPosition	SamRposition	SamRlog2FC	SamRq-value(%)
212789 at	NCAPD3	ENSG00000151503	5.58	0	1	96	1.23	0.00
204560 at	FKBP5	ENSG00000096060	5.08	0	2	30	2.04	0.00
200862 at	DHCR24	ENSG00000116133	3.38	0	3	91	1.14	0.00
205876 at	LIFR	ENSG00000113594	4.24	4.72E-307	5	374	0.68	0.89
219555 s at	CENPN	ENSG00000166451	5.09	2.12E-295	6	29	1.93	0.00
222118 at	CENPN	ENSG00000166451	5.09	2.12E-295	6	11	2.58	0.00
218211 s at	MLPH	ENSG00000115648	2.69	1.29E-282	7	913	0.47	3.09
203455 s at	SAT1	ENSG00000130066	2.93	1.15E-193	10	146	0.95	0.32
210592 s at	SAT1	ENSG00000130066	2.93	1.15E-193	10	161	0.91	0.32
213988 s at	SAT1	ENSG00000130066	2.93	1.15E-193	10	148	0.95	0.32

The list of identifiers and their associated genes extracted from the NGS dataset using DESeq analysis and put to the sscMap. We established where these genes were located in full list (Table S3) of statistically differentially expressed genes returned by the SamR analysis on the microarray dataset. All these genes lay within a SamR reported FDR of 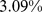. Table S4 also contains the signed ranks of these 10 probesetIDs in the 6 instances of reference profiles for cotinine.

**Figure 3:**
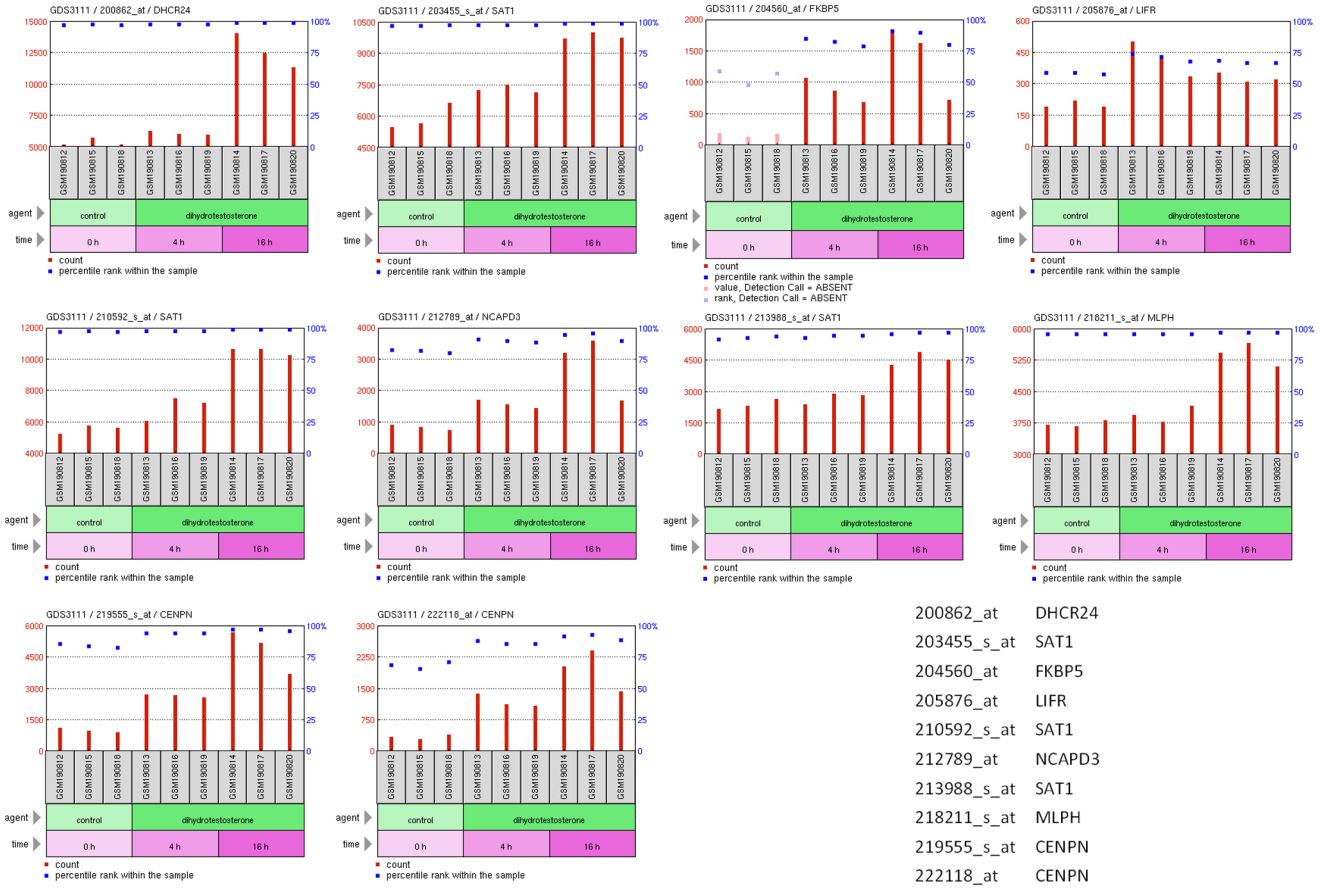
NGS signature genes explored in Microarray study. The set of genes utilised in the NGS gene signature for sscMap are explored in the GEO Dataset Browser with the Wang et al microarray dataset.

Utilising the microarray dataset from Wang et al. that had 0 hr, 4 hr and 16 hr treated LNCaP cells, with the GEO dataset browser we can extract the graphs for each of the ten gene identifiers in Table 3, all of which depict an increase in expression after 16 hrs treatment in the microarray dataset (Figure 3). The microarray analysis of the U133A plus 2 arrays by SamR revealed a list of differentially expressed genes that were extracted by a stringent threshold after genefilter removed half of the genes that had the lowest variance. The U133A Plus 2 array after genefilter were reduced from 54,675 probes to 27,337. From here, we used the 0 hr versus 16 hr data to analyse by SamR. Utilising the delta table we selected the first median FDR value closest to 0.05, ie, a median FDR threshold of 0.068, which gave a delta value of 0.0841, as our stringency threshold. This returned a list of 1313 genes as significant. We then examined if the genes extracted from NGS for sscMap would have been within this stringent list of U133A Plus 2 identifiers (See Table S3 for the full list of differentially expressed genes from the EdgeR analysis). We found all ten genes present, and present within an FDR of 0.0309. Three of the NGS top ten Probesets are within the top 30 most differentially expressed by U133A Plus 2 array, two CENPN identifiers and one FKBP5 identifier (see Table 3), which are viewed with the GEO Dataset Browser in Figure 3.

**Figure 4:**
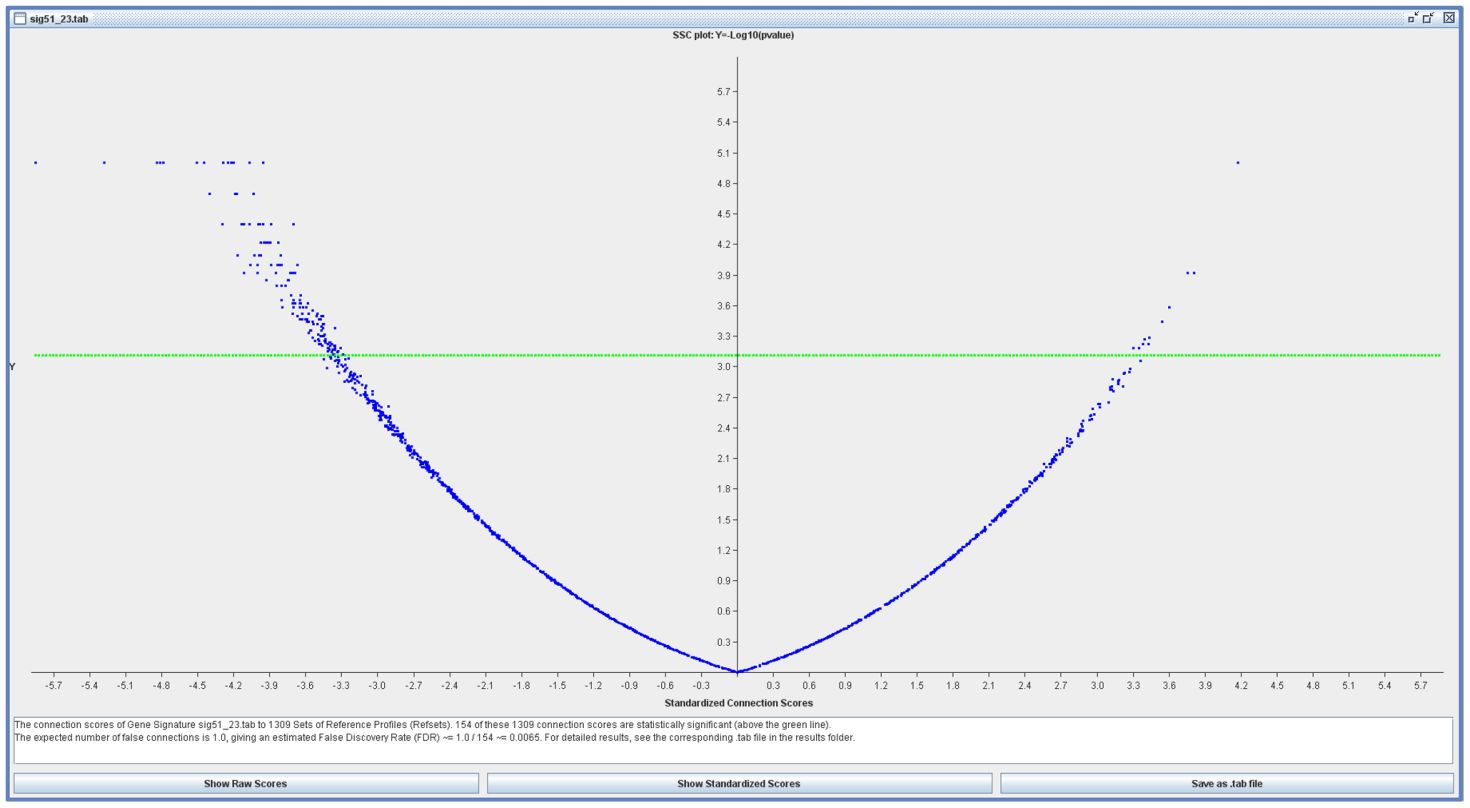
sscMap output for the signature from the Microarray dataset. Distribution of candidate compounds that may enhance (right side) or suppress (left side) the phenotype of the Microarray study.

The top genes from the microarray analysis were extracted and put to connectivity mapping with the same procedures as above. In order to achieve an FDR of 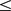
1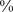, the top 51 genes were extracted and used in mapAffy which returned 23 Affymetrix HG-U133A probeset IDs. These 23 Affy probeset IDs composed the gene signature from the microarray dataset, and they are listed in Table 4 together with the corresponding results from DESeq analysis on the NGS dataset. This gene signature with 23 Affymetrix IDs and their associated expression status were put to the sscMap for perturbation analysis. The results for this gene signature (Figure 4) were that 154 compounds were declared significant with 64 of them having full perturbation stability. The top ranking compounds that would potentially enhance the phenotype were furazolidone with a setsize of 4 and a setscore of 0.344 and PF-00539745-00 with a setsize of 3 and a setscore of 0.341, and those that would potentially suppress the phenotype were indometacin with a setsize of 8 and a setscore of −0.227 and cotinine with a setsize of 6 and a setscore of −0.377.

**Table 4:** The gene signature from the microarray dataset using SamR analysis.

ProbeSetID	GeneSymbol	log2FoldChange	SamR-q-value(%)	SamRposition	DESeqPosition	log2Ratio	adjustedPvalue
209854 s at	KLK2	3.84	0	1	15	3.18	5.77E-160
210339 s at	KLK2	3.48	0	2	15	3.18	5.77E-160
205041 s at	ORM1/ORM2	3.78	0	3	NA	NA	NA
211689 s at	TMPRSS2	3.12	0	4	18	2.26	8.87E-130
222118 at	CENPN	2.58	0	11	6	5.09	2.12E-295
217875 s at	PMEPA1	2.38	0	13	142	2.99	1.4E-30
205862 at	GREB1	2.36	0	14	1356	1.00	0.000149717
219049 at	CSGALNACT1	2.21	0	15	1061	1.81	0.0000119
205102 at	TMPRSS2	2.07	0	16	NA	NA	NA
204583 x at	KLK3	1.96	0	20	31	1.81	1.14E-102
204582 s at	KLK3	2.02	0	21	31	1.81	1.14E-102
209706 at	NKX3-1	1.95	0	22	36	2.49	2.59E-90
219555 s at	CENPN	1.93	0	29	6	5.09	2.12E-295
204560 at	FKBP5	2.04	0	30	2	5.08	0
203196 at	ABCC4	1.69	0	33	23	2.85	5.94E-117
221584 s at	KCNMA1	1.57	0	35	85	2.16	2.31E-43
204897 at	PTGER4	1.62	0	37	671	3.53	0.000000015
211548 s at	HPGD	1.60	0	38	185	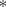 DIV0!	3.19E-25
220014 at	PRR16	1.50	0	44	NA	NA	NA
219476 at	C1orf116	1.47	0	46	96	1.94	5.44E-39
203180 at	ALDH1A3	1.46	0	48	668	1.29	1.44E-08
210787 s at	CAMKK2	1.42	0	49	NA	NA	NA
201110 s at	THBS1	1.38	0	51	NA	NA	NA

The list of identifiers and their associated genes extracted from the microarray using SamR analysis and put to the sscMap. 18 out these 23 gene identifiers are also identified as differentially expressed genes (DEGs) by the DESeq analysis on the NGS dataset (Table S1). NA indicates that the corresponding gene was not returned as DEG by DESeq and hence is not found in Table S1. Expression fold change is defined as ratio (stimulated/unstimulated). 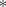 Note that Dseq reported 0 expression for this gene in the unstimulated state, hence ratio(stimulated/unstimulated) and logratio are not defined. Table S5 also contains the signed ranks of these 23 probesetIDs in the 6 instances of reference profiles for cotinine.

We contrasted those 64 compounds with full perturbation stability from the microarray dataset against those 122 compounds from RNA-Seq dataset, the overlap between these two lists of compounds is 18, which are itemized in Table 5. We also carried out a hypergeometric test to gauge the statistical significance of having 18 overlapping drugs between two lists (respectively of 64 and 122 drugs) on a population of 1309 drugs from the sscMap database. The result is highly significant, with a p-value of 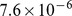. This result indicated a high level of compatibility between the two different technologies, at least for the purpose gene expression connectivity mapping to establish/reveal the biological connections. The RNA-Seq gene signature generally yielded setscores with higher magnitude compared to that of the microarray signature. In each case of these 18 compounds, there was one hundred percent concordance in the direction of their setscores declaring what role (enhancing or suppressing) they would play in influencing the phenotype represented by the signatures. Of these 18 overlapping compounds cotinine was ranked the top in both the NGS and microarray based results, and we chose this compound for laboratory validation of its inhibitory effects on the proliferative phenotype. For the NGS gene signature, Table S4 contains the signed ranks of its 10 probesetIDs in the 6 instances of reference profiles of cotinine, whereas similar information can be found in Table S5, for the 23 probesetIDs of the microarray gene signature.

**Table 5:** Compounds declared significant between both technologies that had full perturbation stability.

refsetname	setsize	queryName	queryLength	setscore	sig	Per	refsetname	setsize	queryName	queryLength	setscore	sig	Per
cotinine	6	RNA-seq	10	−0.598	1	1	cotinine	6	Microarray	23	−0.377	1	1
morantel	5	RNA-seq	10	−0.557	1	1	morantel	5	Microarray	23	−0.366	1	1
tobramycin	4	RNA-seq	10	−0.671	1	1	chlorphenesin	4	Microarray	23	−0.398	1	1
trioxysalen	4	RNA-seq	10	−0.658	1	1	trioxysalen	4	Microarray	23	−0.383	1	1
pentoxyverine	4	RNA-seq	10	−0.601	1	1	trimetazidine	4	Microarray	23	−0.370	1	1
levamisole	4	RNA-seq	10	−0.569	1	1	pentoxyverine	4	Microarray	23	−0.369	1	1
trimetazidine	4	RNA-seq	10	−0.552	1	1	levamisole	4	Microarray	23	−0.356	1	1
chlorphenesin	4	RNA-seq	10	−0.548	1	1	lysergol	4	Microarray	23	−0.349	1	1
oxprenolol	4	RNA-seq	10	−0.535	1	1	tobramycin	4	Microarray	23	−0.348	1	1
zomepirac	4	RNA-seq	10	−0.533	1	1	oxprenolol	4	Microarray	23	−0.336	1	1
lysergol	4	RNA-seq	10	−0.505	1	1	zomepirac	4	Microarray	23	−0.330	1	1
fosfosal	4	RNA-seq	10	−0.428	1	1	fosfosal	4	Microarray	23	−0.280	1	1
sertaconazole	4	RNA-seq	10	−0.411	1	1	sertaconazole	4	Microarray	23	−0.256	1	1
abamectin	4	RNA-seq	10	−0.392	1	1	abamectin	4	Microarray	23	−0.245	1	1
saquinavir	4	RNA-seq	10	−0.359	1	1	saquinavir	4	Microarray	23	−0.243	1	1
ipratropium bromide	3	RNA-seq	10	−0.564	1	1	ipratropium bromide	3	Microarray	23	−0.365	1	1
furazolidone	4	RNA-seq	10	0.602	1	1	furazolidone	4	Microarray	23	0.344	1	1
5186223	1	RNA-seq	10	0.701	1	1	5186223	1	Microarray	23	0.504	1	1

The list of compounds that overlapped between the two technologies, which was 18 out of a possible 64. 16 of the 18 compounds were candidates that would potentially suppress the phenotype. queryLength is the number of genes included in the query gene signature. refset is the set of reference profiles for a compound in the cmap database; Setsize is the size of the set of Reference Profiles for that compound in the cmap core database. sig = 1 indicates the connection score is statistically significant; Per = 1 means that the connection has full perturbation stability.

### GeneCodis Analysis

GeneCodis analysis was conducted on the list of differentially expressed genes from the NGS dataset gene signature (Table 3) and the microarray dataset gene signature (Table 4). Twenty-two sets of processes were significantly enriched, with a corrected hypergeometric p-value of less than 0.05 (Table 6). Thirteen out of these twenty-two significantly enriched processes involved overlapping genes from the NGS signature and the microarray signature.

**Table 6:** GeneCodis analysis with twenty-two sets of processes that were significantly enriched.

Items	NumGenes	ListSize	Ref-Support	Ref-Size	pvalue	Corrected pvalue	NGS	NGS-Microarray	Microarray
GO:0005886: plasma membrane (CC)	7	24	3575	34208	0.009427	0.021997	LIFR	–	C1orf116, PTGER4, KCNMA1, PMEPA1, ABCC4, TMPRSS2
Transcription Factor: V$ER Q6 01	3	24	202	34208	0.000375	0.003279	LIFR	–	CAMKK2, GREB1
GO:0016020: membrane (CC),GO:0005886: plasma membrane (CC)	3	24	603	34208	0.008374	0.020936	–	–	KCNMA1, ABCC4, TMPRSS2
GO:0003824: catalytic activity (MF)	3	24	371	34208	0.002163	0.009461	–	–	KLK3, KLK2, HPGD
GO:0006508: proteolysis (BP),GO:0008233: peptidase activity (MF), (InterPro) IPR001314: Peptidase S1A chymotrypsin-type, (InterPro) IPR001254: Peptidase S1/S6, chymotrypsin/Hap, GO:0004252: serine-type endopeptidase activity (MF)	3	24	97	34208	0.000043	0.000500	–	–	KLK3, KLK2, TMPRSS2
GO:0005576: extracellular region (CC)	5	24	1913	34208	0.009477	0.020731	LIFR	–	KLK3, THBS1, ORM2, TMPRSS2
GO:0016021: integral to membrane (CC)	7	24	4400	34208	0.027478	0.043715	DHCR24	–	PTGER4, KCNMA1, PMEPA1, ABCC4, GREB1, CSGALNACT1
GO:0007596: blood coagulation (BP),GO:0030168: platelet activation (BP)	3	24	218	34208	0.000468	0.003276	–	–	THBS1, KCNMA1, ABCC4
GO:0005634: nucleus (CC),GO:0005829: cytosol (CC)	3	24	863	34208	0.021829	0.036382	DHCR24	CENPN	HPGD
GO:0005737: cytoplasm (CC)	9	24	5302	34208	0.007291	0.023198	SAT1, DHCR24, MLPH	FKBP5	C1orf116, ALDH1A3, CAMKK2, HPGD, TMPRSS2
Transcription Factor: V$MYC Q2	3	24	741	34208	0.014602	0.028393	–	FKBP5	CAMKK2, ABCC4
GO:0016020: membrane (CC),Transcription Factor: V$FOXO4 01	3	24	306	34208	0.001248	0.006240	–	FKBP5	GREB1, TMPRSS2
GO:0005737: cytoplasm (CC); Transcription Factor: V$SP1 Q6	3	24	862	34208	0.021763	0.038085	MLPH	FKBP5	HPGD
GO:0005634: nucleus (CC),Transcription Factor: V$E12 Q6	3	24	587	34208	0.007781	0.020949	–	FKBP5	HPGD, NKX3-1
GO:0016020: membrane (CC)	7	24	4065	34208	0.018458	0.034002	DHCR24	FKBP5	KCNMA1, ABCC4, GREB1, TMPRSS2, CSGALNACT1
GO:0016020: membrane (CC), Transcription Factor: V$E12 Q6	3	24	401	34208	0.002695	0.009433	–	FKBP5	KCNMA1, GREB1
Transcription Factors: V$E12 Q6,V 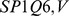 CHX10 01	3	24	65	34208	0.000013	0.000451	–	FKBP5	KCNMA1, HPGD
Transcription Factor: V$E12 Q6	5	24	1805	34208	0.007455	0.021744	–	FKBP5	KCNMA1, HPGD, GREB1, NKX3-1
GO:0005634: nucleus (CC),Transcription Factor: V$NFY Q6 01	3	24	401	34208	0.002695	0.009433	DHCR24	FKBP5	NKX3-1
GO:0005488: binding (MF)	3	24	731	34208	0.014083	0.028993	NCAPD3	FKBP5	ORM2
GO:0016020: membrane (CC),Transcription Factor: V$LEF1 Q2,GO:0005737: cytoplasm (CC)	3	24	90	34208	0.000034	0.000599	DHCR24	FKBP5	TMPRSS2
GO:0016020: membrane (CC),Transcription Factor: V$NFAT Q4 01	3	24	286	34208	0.001028	0.005995	–	–	KCNMA1, GREB1, TMPRSS2

GeneCodis analysis for both signatures. pvalue and corrected pvalue are the hypergeometric pvalues with Ref standing for reference.

### Laboratory Confirmation

Cotinine, the nicotine metabolite, is commonly found in tobacco and is an inhibitor of 3 alpha- hydroxysteroid dehydrogenase (HSD) which converts DHT to 3 alpha-androstanediol. Stimulation of LNCaP cells with DHT would selectively activate this androgen related pathway in these cells, causing an increase in proliferation rates, while pre-treatment with cotinine would clearly block the activation of this pathway. The appearance of cotinine as the top candidate which could suppress the phenotype of DHT stimulation in the androgen-sensitive LNCaP cell line highlight the reliable nature of the connectivity mapping procedure given that the dataset was obtained from androgen stimulated cells.

**Figure 5:**
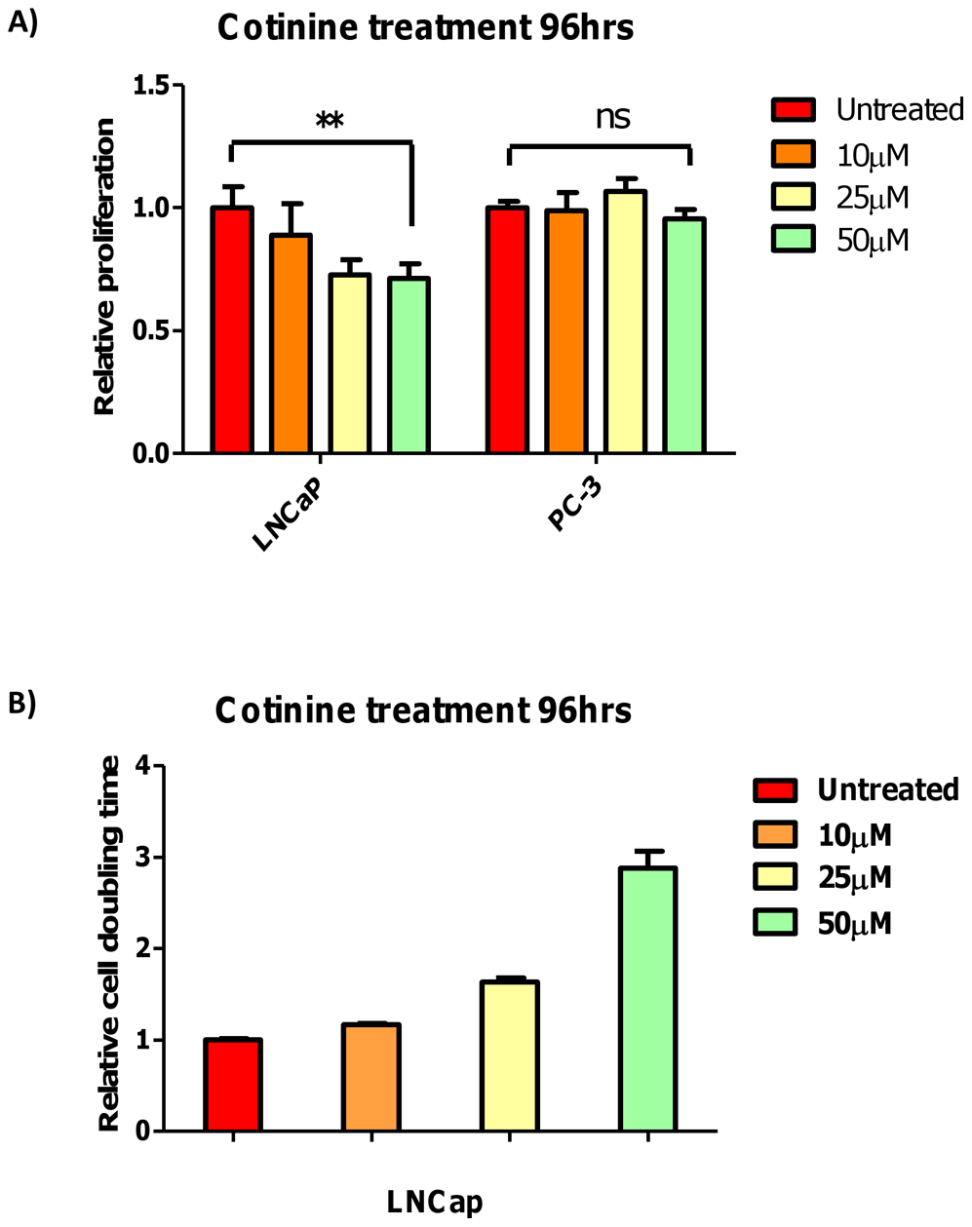
Cotinine was the top candidate compound to suppress the proliferative phenotype. Validation of cotinine as the top candidate to suppress cell proliferation phenotype induced via androgen pathway. A, Cells in 6 well plate format were treated with vehicle or various doses of cotinine for 96 hours. Total viable cell numbers were counted by haemocytometer. Cell counts are represented as relative to the untreated control value. B, Cells were treated with indicated doses of cotinine and seeded in xCELLigence 16 well E-plates. Real-time analysis of cell doubling rates was recorded and rate of doubling between 72–96 hours was plotted relative to untreated control using the system software. The experiment was repeated three times with similar results. ** denotes 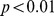 using unpaired two-tailed t-test.

To biologically test the relevance of the top candidate drug we carried out cell proliferation, viability and cell doubling assays either in the absence or presence of cotinine at doses which have been used in the literature on the androgen dependent LNCaP cells and compared these results with the androgen independent PC3 cells. The PC3 cells were chosen as this cell line is no longer dependent on the pathways which cotinine will inhibit, therefore unlike the LNCaP cells, the proliferation of these cells should not be affected by cotinine. Indeed, we found that the compound significantly inhibited overall cell proliferation of the LNCaP cell line but not that of the PC3 at the doses used (Figure 5A). The reduction in cell proliferation was attributed to an increased cell doubling time following cotinine treatment (Figure 5B), which would reduce overall cell numbers in the treated group. These findings may be even more significant given that this was carried out in the absence of any external androgen stimulation as we would assume that the stimulation of these cells with DHT would further activate the pathway, causing increased proliferation, which could then be blocked by the cotinine.

## Discussion

The phenotype of androgen stimulation in androgen-sensitive cell lines has long been known to result in increased proliferation rates through, in part, androgen receptor translocation to the nucleus and activation of transcription resulting in cell growth, although the complete mechanism remains to be characterised. The results of this study demonstrated a strong overlap of candidate compounds which could be used to influence the phenotype in a laboratory setting. We were able to show the same compound could be detected from both platforms that represented the best opportunity to suppress the phenotype, which in this case is androgen driven cell proliferation. Tested in the laboratory, cotinine, was shown to be able to inhibit proliferation in LNCaP cells but not in the PC-3 controls. These effects were found to be mainly due to decreased proliferation and cell division rates (Figure 5) and not due to increased cell death in the treated samples as no evidence of increased numbers of non-viable cells were found (data not shown). The full effects on the transcriptome of LNCaP cells following cotinine treatment either in the absence or presence of an external androgen stimulant, such as DHT or R1881, would no doubt give further insights into the mechanism of proliferation inhibition.

There is scientific precedence for using cotinine as an anti-proliferative drug in a hormone sensitive setting. In an in vitro study choriocarcinoma cells, which are germ cell tumour cells arising in the testis or ovary, were exposed to increasing concentrations of cotinine and examination of serum hormone levels was carried out. Addition of cotinine inhibited estradiol accumulation in choriocarcinoma cells at micromolar concentrations [40]. It was also noted that exposure to cotinine in placental microsomes from term females gave a similar response as the drug aminoglutethimide, which is an inhibitor of aromatase that is used clinically in the hormonal therapy of metastatic breast cancer. The functional similarity of these compounds may be explained by the substantial chemical structural similarities that they share. Another study also confirmed that exposure to cotinine functioned in a competitive manner to inhibit testosterone biosynthesis in rat cells [41]. In a recent clinical study of healthy females it was concluded that smoke exposure was associated with lower than normal median steroid hormone concentrations. In general non-smokers tended to have higher median hormone concentrations than smokers and passive smokers. In the particular case of androgens, when classified by serum cotinine levels, it was noted that the median concentrations of testosterone, cortisol, and androgen steroid hormone analytes were highest in non-smokers who would have lower cotinine serum levels compared to smokers [42].

The p38 inhibitor SB-202190, which was another candidate compound from the connectivity mapping list, is a highly selective and cell permeable inhibitor which has also been shown to specifically inhibit the androgen pathway in LNCaP cell lines. P38 is a signalling protein and a member of the mitogen-activated protein kinase (MAPK) pathway. MAPK signalling has long been associated with cancer initiation and progression in a number of disease settings and indeed the role of P38 has been well illustrated in the progression of prostate cancer [43]. There have been numerous investigations into the role of P38 in androgen responsive gene activation and prostate cancer progression. Pretreatment of LNCaP cells with SB-202190 could inhibit androgen receptor mediated activation of the PSA promotor by IL-6, a factor which is known to promote prostate cancer growth. The same group also noted that treatment of LNCaP cells with either IL-6 or DHT caused nuclear translocation of the androgen receptor another key process involved in androgen responsiveness, which would be halted by SB-202190 pre-treatment [44]. Inhibition of the P38 pathway in this setting would therefore reverse the effects observed with DHT stimulation again confirming the results of the connectivity mapping.

Another prostate cancer study in Oncogene using SB-202190 found that formation of fibroblast growth factor (FGF) induced actin stress fibres could be abolished using this inhibitor. These cytoskeletal changes are of importance in the progression of the disease and these factors are known to be over-expressed in human prostate cancer. This work highlights the p38-dependent nature of this progression, again confirming the validity of SB-202190 on the connectivity list [45].

The other compounds on the list, belonging to the NSAID family, are of interest at present given the recent numerous publications linking this class of drugs with anti-cancer effects. There is substantial evidence to suggest that regular use of NSAID's can reduce an individual's risk of developing a number of cancers. Recent studies have shown that NSAID's can be beneficial as they can induce cell death and inhibit epidermal growth factor receptor signalling via the MAPK pathway in colon cancer [46]. Another study again highlighted the pro apoptotic effects of NSAID treatment via inhibition of protein synthesis in colon cancer and a number of other cancer cell types [47]. Their use has again been in the clinical spotlight with the recent publication re-opening the debate of their benefits as an adjuvant therapy and also the anti-cancer properties of NSAID's in patients with Lynch Syndrome were published recently [48].

GeneCodis highlighted a number of overlapping sets of processes which were identified by either the NGS or microarray analysis or both sets of data (Table 6). Given the high concordance of the top differentially expressed genes identified using both datasets it was unsurprising to find significant enrichment of 13 out of 22 of the processes driven by NGS/microarray overlapping genes using the GeneCodis analysis. The overlapping nature of the signatures and processes can also be attributed to the fact that both datasets are derived from comparison of DHT stimulated or un-stimulated androgen responsive prostate cancer cells. The resulting genes that were differentially expressed would all have been due to activation of the androgen pathway and the candidate compounds that were top of the connectivity mapping would specifically block one of these components. These findings further confirm the robustness of the connectivity mapping technique in identifying candidate compounds to inhibit a targeted phenotype.

One point worth noting is that the two datasets used have limited sample size, particularly for the microarray dataset 3 vs 3 samples is small, although for the RNA-seq data set 3 vs 4 samples is moderate considering the current literature norm. On the other hand, the two datasets were both from cell line based experiments; Unlike human patient samples, cell line experiments are much less heterogenous and hence less demanding on sample sizes. That said, the important safeguard in our analysis is that we applied very stringent criteria in differential expression analysis to exert effective control of false discoveries. Another compensating factor for the small sample sizes is that for connectivity mapping purposes, we often only need a small set of very significant top DEGs (differentially expressed genes) to establish/reveal the biological connections, and thus the demand on sample sizes can be alleviated to some extent. A noisy factor that may affect the comparison between the two datasets is that although the two studies are both related to prostate cancer, the study goal and process are slightly different. In spite of this, the highly significant overlap between the two connectivity mapping exercises is really encouraging for such integrative approach in future studies.

The results we presented in this work points to a promising prospect of integrating RNA-seq data with connectivity mapping. But it is also important point out the limitation of this approach. Reference profiles in the current connectivity mapping databases were all built on microarray technology, which may have been limited by its dynamic range, sensitivity and potential bias towards the pre-defined probes. RNA-seq on the other hand provides more comprehensive and thorough assessment to the coding region. It is inevitable that there will be information loss when mapping RNA-seq data to the array-based reference profiles. For example, of the top 10 transcript IDs retrieved from the DESeq analysis in Table 1, 3 of them could not be mapped to Affymetrix HG-U133A IDs used in the reference profiles. Ultimately when the cost of RNA-seq drops to comparable level with microarray, it will become more realistic to re-build the reference expression profiles purely based on the new NGS technology, which in turn probably requires a more radical change of the mathematical framework currently employed in the array based connectivity mapping. In the meantime, the integration attempt we made here contrasted RNA-Seq extrapolated signatures with those of a traditional microarray based approach in order to bridge impending costs in establishing compound reference profiles. Emergent alternative compound analysis such as the novel L1000 gene expression analysis offers an attractive platform that has 1000 mRNA transcripts per Luminex well. It allows for the detection of up to 100 transcripts in many thousands of samples by a flexible and cost effective multiplex ligation-mediated amplication on a specialised Luminex FlexMAP [49].

The choice of study carried out here was of a well known RNA-Seq dataset with which we analysed with an established pipeline in order to retrieve a list of differentially expressed genes that we could contrast against a published microarray dataset of similar design. We first attained a signature with an appropriate false discovery rate, then the gene signature was perturbed to checked the robustness of discovered connections to sscMap compounds. This allowed us to rank the candidate compounds by their perturbation stability and thus have increased confidence in their ability to alter the phenotype. The biological outputs from the two technologies tell a similar story because of the common underlying phenotype being studied. In order to make the most of the sensitivity of the RNA-Seq technology, the sequence mapping tools in the pipeline need to be considered along with appropriate algorithms for differential expression. Su et al. noted that the choice of aligners between bowtie, SOAP2 and BWA had a strong concordance of 98 percent, coupled with the fact that Kvam et al. noted that the choice of differential expression analysis tool may vary slightly although edgeR and DESeq performed similarly [15], [17]. As newer NGS analysis software become available coupled with a decrease in cost of RNA-Seq, future studies using these techniques will inevitably afford larger sample sizes with sensitivity and power furthered increased. The flexible and extensible existing connectivity map software together with new and emerging tools in connectivity mapping analysis, such as DvD [50] which utilize Gene Expression Omnibus and Array Express databases for drug repurposing will undoubtedly become a valuable resource for discovering candidate therapeutics in cancer research.

## Supporting Information

Table S1 **A comprehensive list of differentially expressed genes from DESeq analysis on the RNA-seq dataset.** Genes are primarily sorted by p-value in ascending order, and sub-sorted by the absolute value of log2ratio in descending order in case of equal p-values. All genes with adjusted p value less than 0.05 are listed here.
(XLSX)

Table S2 **A comprehensive list of differentially expressed genes from EdgeR analysis on the RNA-seq dataset.** Genes are primarily sorted by p-value in ascending order, and sub-sorted by the absolute value of log2ratio in descending order in case of equal p-values. All genes with adjusted p value less than 0.05 are listed here.
(XLSX)

Table S3 **A comprehensive list of differentially expressed genes from SamR analysis on the microarray dataset.** Genes are primarily sorted by q-value in ascending order, and sub-sorted by the absolute value of d scores of SamR in descending order in case of equal q-values. All genes listed here have a q value less than the cutoff 0.0683, which was the FDR threshold closest to 0.05 in choosing the delta value in the SamR analysis.
(XLSX)

Table S4 **The gene signature obtained from DESeq analysis on the NGS dataset.** Also included in this table are the signed ranks of these 10 probesetIDs in the six instances of reference profiles for cotinine. The magnitude of the rank indicates the importance of the gene in that reference profile; a minus sign indicates that the gene was down-regulated in the drug treatment experiment.
(XLSX)

Table S5 **The gene signature obtained from SamR analysis on the microarray dataset.** Also included in this table are the signed ranks of these 23 probesetIDs in the six instances of reference profiles for cotinine. The magnitude of the rank indicates the importance of the gene in that reference profile; a minus sign indicates that the gene was down-regulated in the drug treatment experiment.
(XLSX)

## References

[1] Garber M, Grabherr MG, Guttman M, Trapnell C (2011) Computational methods for transcriptome annotation and quantification using rna-seq. Nat Meth 8: 469–477. 10.1038/nmeth.1613 21623353

[2] Oshlack A, Robinson M, Young M (2010) From rna-seq reads to differential expression results. Genome Biology 11: 220. 21176179 10.1186/gb-2010-11-12-220 PMC3046478

[3] Ding L, Wendl MC, Koboldt DC, Mardis ER (2010) Analysis of next-generation genomic data in cancer: accomplishments and challenges. Human Molecular Genetics 19: R188–R196. 20843826 10.1093/hmg/ddq391 PMC2953747

[4] Denoeud F, Aury JM, Da Silva C, Noel B, Rogier O, et al (2008) Annotating genomes with massive-scale rna sequencing. Genome Biology 9: R175. 19087247 10.1186/gb-2008-9-12-r175 PMC2646279

[5] Cloonan N, Forrest ARR, Kolle G, Gardiner BBA, Faulkner GJ, et al (2008) Stem cell transcriptome profiling via massive-scale mrna sequencing. Nat Meth 5: 613–619. 10.1038/nmeth.1223 18516046

[6] Wang L, Li P, Brutnell TP (2010) Exploring plant transcriptomes using ultra high-throughput sequencing. Briefings in Functional Genomics 9: 118–128. 20130067 10.1093/bfgp/elp057

[7] Ozsolak F, Milos PM (2011) Rna sequencing: advances, challenges and opportunities. Nat Rev Genet 12: 87–98. 21191423 10.1038/nrg2934 PMC3031867

[8] Roberts A, Trapnell C, Donaghey J, Rinn J, Pachter L (2011) Improving rna-seq expression estimates by correcting for fragment bias. Genome Biology 12: R22. 21410973 10.1186/gb-2011-12-3-r22 PMC3129672

[9] Glenn TC (2011) Field guide to next-generation dna sequencers. Molecular Ecology 11: 759–769. 10.1111/j.1755-0998.2011.03024.x 21592312

[10] Langmead B, Trapnell C, Pop M, Salzberg S (2009) Ultrafast and memory-efficient alignment of short dna sequences to the human genome. Genome Biology 10: R25. 19261174 10.1186/gb-2009-10-3-r25 PMC2690996

[11] Rumble SM, Lacroute P, Dalca AV, Fiume M, Sidow A, et al (2009) Shrimp: Accurate mapping of short color-space reads. PLoS Comput Biol 5: e1000386. 19461883 10.1371/journal.pcbi.1000386 PMC2678294

[12] Li R, Li Y, Kristiansen K, Wang J (2008) Soap: short oligonucleotide alignment program. Bioinformatics 24: 713–714. 18227114 10.1093/bioinformatics/btn025

[13] Li H, Durbin R (2009) Fast and accurate short read alignment with burrows-wheeler transform. Bioinformatics 25: 1754–1760. 19451168 10.1093/bioinformatics/btp324 PMC2705234

[14] Li H, Homer N (2010) A survey of sequence alignment algorithms for next-generation sequencing. Briefings in Bioinformatics 11: 473–483. 20460430 10.1093/bib/bbq015 PMC2943993

[15] Kvam VM, Liu P, Si Y (2012) A comparison of statistical methods for detecting differentially expressed genes from rna-seq data. American Journal of Botany 99: 248–256. 22268221 10.3732/ajb.1100340

[16] Marioni JC, Mason CE, Mane SM, Stephens M, Gilad Y (2008) Rna-seq: An assessment of technical reproducibility and comparison with gene expression arrays. Genome Research 18: 1509–1517. 18550803 10.1101/gr.079558.108 PMC2527709

[17] Su Z, Li Z, Chen T, Li QZ, Fang H, et al (2011) Comparing next-generation sequencing and microarray technologies in a toxicological study of the effects of aristolochic acid on rat kidneys. Chem Res Toxicol 24: 1486–1493. 21834575 10.1021/tx200103b

[18] Goncalves A, Tikhonov A, Brazma A, Kapushesky M (2011) A pipeline for rna-seq data processing and quality assessment. Bioinformatics 27(6): 867–9. 21233166 10.1093/bioinformatics/btr012 PMC3051320

[19] Lamb J, Crawford ED, Peck D, Modell JW, Blat IC, et al (2006) The connectivity map: Using gene-expression signatures to connect small molecules, genes, and disease. Science 313: 1929–1935. 17008526 10.1126/science.1132939

[20] Iorio F, Bosotti R, Scacheri E, Belcastro V, Mithbaokar P, et al (2010) Discovery of drug mode of action and drug repositioning from transcriptional responses. Proceedings of the National Academy of Sciences 107: 14621–14626. 10.1073/pnas.1000138107 PMC2930479 20679242

[21] Iskar M, Campillos M, Kuhn M, Jensen LJ, van Noort V, et al (2010) Drug-induced regulation of target expression. PLoS Comput Biol 6: e1000925. 20838579 10.1371/journal.pcbi.1000925 PMC2936514

[22] Zhang SD, Gant TW (2009) sscmap: An extensible java application for connecting small-molecule drugs using gene-expression signatures. BMC Bioinformatics 10: 236. 19646231 10.1186/1471-2105-10-236 PMC2732627

[23] Zhang SD, Gant TW (2008) A simple and robust method for connecting small-molecule drugs using gene-expression signatures. BMC Bioinformatics 9: 258. 18518950 10.1186/1471-2105-9-258 PMC2464610

[24] McArt DG, Zhang SD (2011) Identification of candidate small-molecule therapeutics to cancer by gene-signature perturbation in connectivity mapping. PLoS ONE 6: e16382. 21305029 10.1371/journal.pone.0016382 PMC3031567

[25] Iorio F, Rittman T, Ge H, Menden M, Saez-Rodriguez J (2013) Transcriptional data: a new gateway to drug repositioning? Drug Discovery Today 18: 350–357. 22897878 10.1016/j.drudis.2012.07.014 PMC3625109

[26] Li H, Lovci MT, Kwon YS, Rosenfeld MG, Fu XD, et al (2008) Determination of tag density required for digital transcriptome analysis: Application to an androgen-sensitive prostate cancer model. Proceedings of the National Academy of Sciences 105: 20179–20184. 10.1073/pnas.0807121105 PMC2603435 19088194

[27] Wang Q, Li W, Liu XS, Carroll JS, Jnne OA, et al (2007) A hierarchical network of transcription factors governs androgen receptor-dependent prostate cancer growth. Molecular Cell 27: 380–392. 17679089 10.1016/j.molcel.2007.05.041 PMC3947890

[28] Kent WJ, Sugnet CW, Furey TS, Roskin KM, Pringle TH, et al (2002) The human genome browser at ucsc. Genome Research 12: 996–1006. 12045153 10.1101/gr.229102 PMC186604

[29] Li H, Handsaker B, Wysoker A, Fennell T, Ruan J, et al (2009) The sequence alignment/map format and samtools. Bioinformatics 25: 2078–2079. 19505943 10.1093/bioinformatics/btp352 PMC2723002

[30] Gentleman RC, Carey VJ, Bates DM, et al (2004) Bioconductor: Open software development for computational biology and bioinformatics. Genome Biology 5: R80. 15461798 10.1186/gb-2004-5-10-r80 PMC545600

[31] Anders S, Huber W (2010) Differential expression analysis for sequence count data. Genome Biology 11: R106. 20979621 10.1186/gb-2010-11-10-r106 PMC3218662

[32] Robinson MD, McCarthy DJ, Smyth GK (2010) edger: a bioconductor package for differential expression analysis of digital gene expression data. Bioinformatics 26: 139–140. 19910308 10.1093/bioinformatics/btp616 PMC2796818

[33] Barrett T, Troup DB, Wilhite SE, Ledoux P, Evangelista C, et al (2011) Ncbi geo: archive for functional genomics data sets-10 years on. Nucleic Acids Research 39: D1005–D1010. 21097893 10.1093/nar/gkq1184 PMC3013736

[34] Edgar R, Domrachev M, Lash AE (2002) Gene expression omnibus: Ncbi gene expression and hybridization array data repository. Nucleic Acids Research 30: 207–210. 11752295 10.1093/nar/30.1.207 PMC99122

[35] Tibshirani R, Chu G, Narasimhan B, Li J. Sam: Significance analysis of microarrays. R Packge versoin 2.0. Available: http://cran.r-project.org/web/packages/samr/index.html. Accessed 2012 Dec 10.

[36] Gautier L, Cope L, Bolstad BM, Irizarry RA (2004) affy-analysis of affymetrix genechip data at the probe level. Bioinformatics 20: 307–315. 14960456 10.1093/bioinformatics/btg405

[37] Gentleman R, Carey V, Huber W, Hahne F. genefilter: methods for filtering genes from microarray experiments. R package version 1.38.0. Available: http://www.bioconductor.org/packages//2.10/bioc/html/genefilter.html. Accessed 2012 Dec 10.

[38] Nogales-Cadenas R, Carmona-Saez P, Vazquez M, Vicente C, Yang X, et al (2009) Genecodis: interpreting gene lists through enrichment analysis and integration of diverse biological information. Nucleic Acids Research 37: W317–W322. 19465387 10.1093/nar/gkp416 PMC2703901

[39] Tabas-Madrid D, Nogales-Cadenas R, Pascual-Montano A (2012) Genecodis3: a non-redundant and modular enrichment analysis tool for functional genomics. Nucleic Acids Research 40: W478–W483. 22573175 10.1093/nar/gks402 PMC3394297

[40] Barbieri RL, Gochberg J, Ryan KJ (1986) Nicotine, cotinine, and anabasine inhibit aromatase in human trophoblast in vitro. The Journal of Clinical Investigation 77: 1727–1733. 3711333 10.1172/JCI112494 PMC370526

[41] Yeh J, Barbieri R, Friedman A (1989) Nicotine and cotinine inhibit rat testis androgen biosynthesis in vitro. J Steroid Biochem 33(4A): 627–630. 2811374 10.1016/0022-4731(89)90051-4

[42] Soldin OP, Makambi KH, Soldin SJ, O’Mara DM (2011) Steroid hormone levels associated with passive and active smoking. Steroids 76: 653–659. 21396948 10.1016/j.steroids.2011.02.042 PMC3635532

[43] Rodrguez-Berriguete G, Fraile B, Martnez-Onsurbe P, Olmedilla G, Paniagua R, et al (2012) Map kinases and prostate cancer. J Signal Transduct 2012: 169170. 22046506 10.1155/2012/169170 PMC3199183

[44] Lin DL, Whitney MC, Yao Z, Keller ET (2001) Interleukin-6 induces androgen responsiveness in prostate cancer cells through up-regulation of androgen receptor expression. Clinical Cancer Research 7: 1773–1781. 11410519

[45] Mehta PB, Robson CN, Neal DE, Leung HY (2001) Keratinocyte growth factor activates p38 mapk to induce stress fibre formation in human prostate du145 cells. Oncogene 20: 5359–5365. 11536048 10.1038/sj.onc.1204688

[46] Tavolari S, Munarini A, Storci G, Laufer S, Chieco P, et al (2012) The decrease of cell membrane fluidity by the non-steroidal anti-inflammatory drug licofelone inhibits epidermal growth factor receptor signalling and triggers apoptosis in hca-7 colon cancer cells. Cancer Letters 321: 187–194. 22343320 10.1016/j.canlet.2012.02.003

[47] Brunelli C, Amici C, Angelini M, Fracassi C, Belardo G, et al (2012) The non-steroidal antiinflammatory drug indomethacin activates the eif2a kinase pkr, causing a translational block in human colorectal cancer cells. Biochemical Journal 443: 379–386. 22268531 10.1042/BJ20111236

[48] Langley RE, Burdett S, Tierney JF, Cafferty F, Parmar MKB, et al (2011) Aspirin and cancer: has aspirin been overlooked as an adjuvant therapy? Br J Cancer 105: 1107–1113. 21847126 10.1038/bjc.2011.289 PMC3208483

[49] Peck D, Crawford E, Ross K, Stegmaier K, Golub T, et al (2006) A method for high-throughput gene expression signature analysis. Genome Biology 7: R61. 16859521 10.1186/gb-2006-7-7-r61 PMC1779561

[50] Pacini C, Iorio F, Gonalves E, Iskar M, Klabunde T, et al (2013) Dvd: An r/cytoscape pipeline for drug repurposing using public repositories of gene expression data. Bioinformatics 29: 132–134. 23129297 10.1093/bioinformatics/bts656 PMC3530913

